# Supplementary material for: A Multifunctional Polyethylene Glycol/Triethoxysilane-Modified Polyurethane Foam Dressing with High Absorbency and Antiadhesion Properties Promotes Diabetic Wound Healing
Source: Int J Mol Sci. 2023 Aug 7;24(15):12506. doi: 10.3390/ijms241512506 (PMC10419382; doi:10.3390/ijms241512506)
Supplement: Supplementary file 1 [file ijms-24-12506-s001.zip › ijms-2475604-supplementary.pdf]

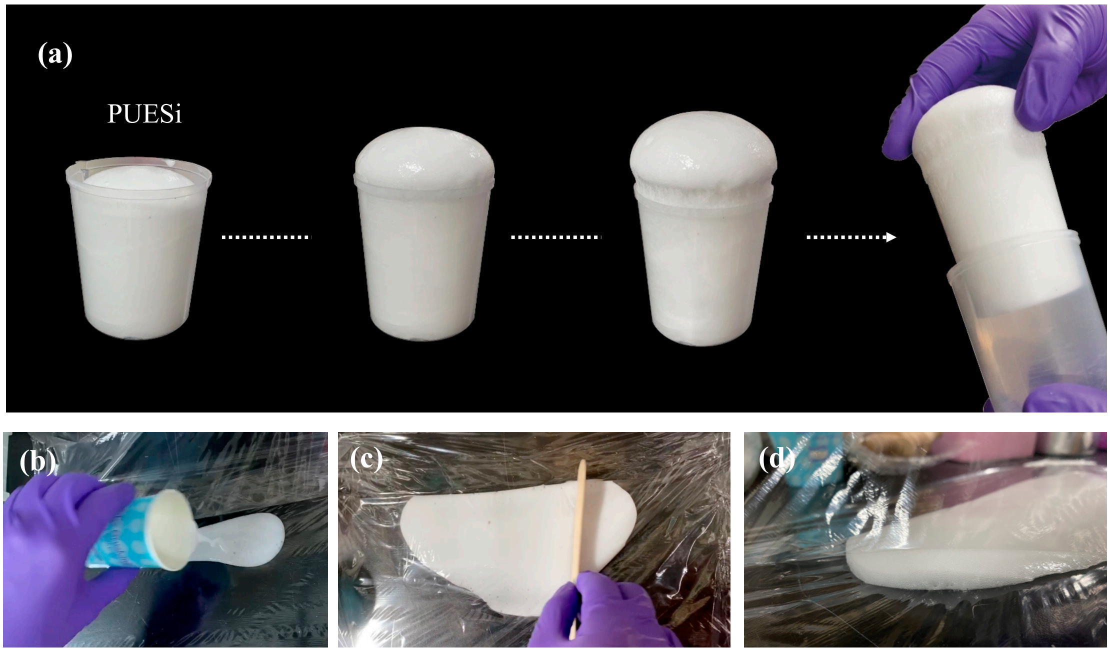


**Figure S1.** Self-foaming reaction (**a**) and foam dressing preparation (**b, c, d**).

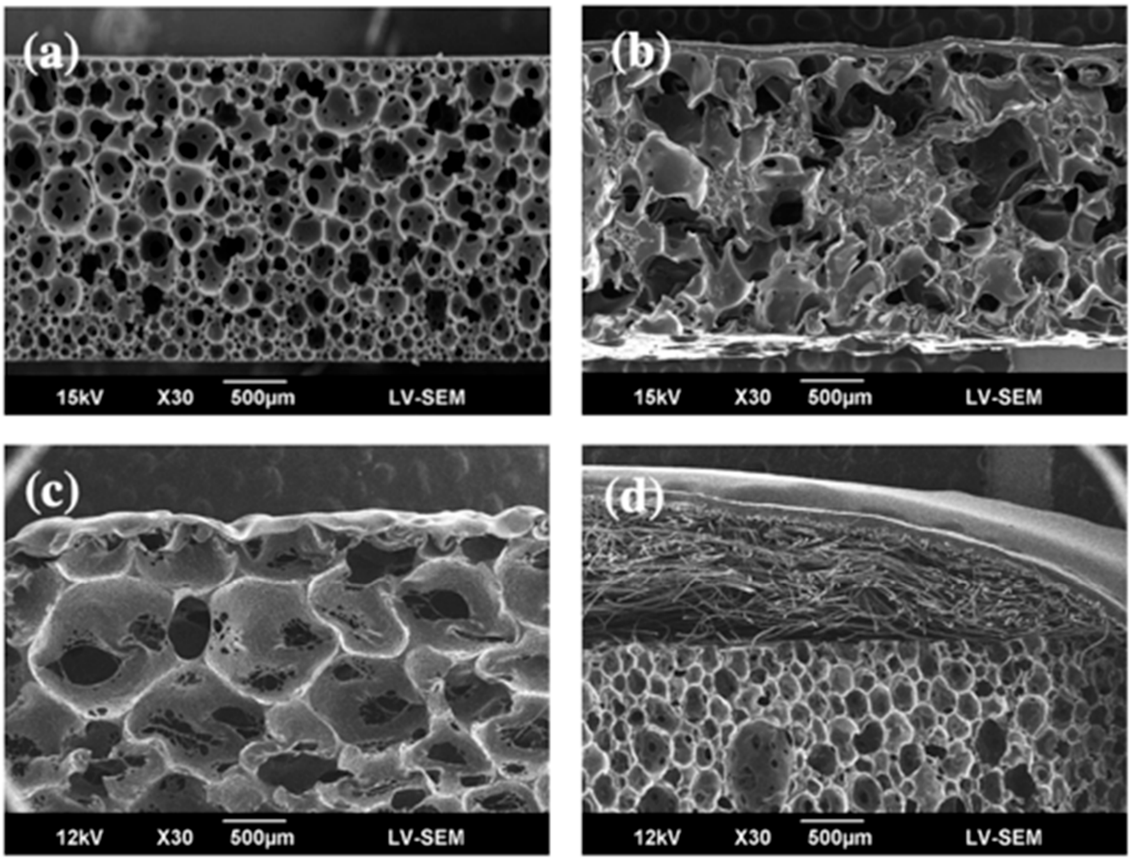


**Figure S2.** Cross-sectional scanning electron microscopy (SEM) images of PUESi (**a**) and the commercial PU foam dressings PolyMem (**b**), Mepilex (**c**) and ConveTec (**d**).
